# Comparison of the clinical efficacy and prognosis of the two techniques for treating partial articular-sided supraspinatus tendon tears under arthroscopy

**DOI:** 10.1186/s12891-024-07634-4

**Published:** 2024-07-05

**Authors:** Li Zhang, Zhanglu Fang, Yin Zhang, Xun Wang, Zheping Hong

**Affiliations:** 1 Department of Sports Medicine, Zhejiang Provincial People’s Hospital (Affiliated People’s Hospital), Hangzhou Medical College, Hangzhou, Zhejiang China; 2 School of Medicine, Zhejiang Chinese Medical University, Hangzhou, Zhejiang China; 3 Institute of Sports Medicine and Osteoarthropathy of Hangzhou Medical College, Hangzhou, Zhejiang China; 4 Center for Rehabilitation Medicine, Department of Orthopedics, Zhejiang Provincial People’s Hospital (Affiliated People’s Hospital), Hangzhou Medical College, Hangzhou, Zhejiang China

**Keywords:** Partial articular-sided supraspinatus tendon tears, Arthroscopic transtendon repair, Arthroscopic full-thickness repair

## Abstract

**Background:**

At present, shoulder arthroscopy is usually used for treatment of rotator cuff injuries. There is still debate over the precise technique of using shoulder arthroscopy to treat partial articular-sided supraspinatus tendon injuries.

**Objective:**

To compare the clinical efficacy of the arthroscopic transtendon repair method and the arthroscopic full-thickness repair method in the treatment of patients with Ellman III partial articular-sided supraspinatus tendon tears and to analyze the influencing factors of postoperative efficacy.

**Study design:**

Cohort study; level of evidence,4.

**Methods:**

A total of 84 partial-thickness rotator cuff tear (PTRCT) patients with Ellman III injuries who underwent surgical treatment in our hospital between January 2017 and January 2020 were selected and divided into the arthroscopic trans-tenon repair group (32 cases) and the arthroscopic full-thickness repair group (52 cases). Shoulder joint pain and functional status were assessed by the Constant score, ASES score and VAS score; shoulder mobility was assessed by measuring shoulder ROM. The clinical outcomes of the two groups of patients were compared, and the factors affecting the postoperative efficacy of the patients were investigated.

**Results:**

All patients were followed up for at least 2 years. The Constant score, ASES score, and VAS score of the two groups of patients were all improved compared with those before surgery, and the differences were statistically significant (*P* < 0.05). There were no significant differences in the Constant score, ASES score, or VAS score between the two groups (*P* > 0.05). The results of binary logistic regression analysis showed that the preoperative ASES score and whether biceps tenotomy was performed were independent risk factors for satisfactory postoperative efficacy (*P* < 0.05).

**Conclusion:**

For patients with Ellman III partial articular-sided supraspinatus tendon tears, the arthroscopic transtendon repair method and the arthroscopic full-thickness repair method can both significantly improve the shoulder pain and function of the patient, but there is no significant difference between the efficacy of the two surgical methods. The preoperative ASES score and whether biceps tenotomy was performed were independent risk factors for satisfactory postoperative efficacy in PTRCT patients with Ellman III injury.

**Supplementary Information:**

The online version contains supplementary material available at 10.1186/s12891-024-07634-4.

## Introduction

With the extension of the human lifespan, the advancement of imaging examination and the development of arthroscopic techniques, the diagnostic rate of partial injury of the supraspinatus tendon has been increasing [1, 2]. With a reported incidence of 17–37%, partial injury of the supraspinatus tendon is a common type of shoulder joint lesion seen in clinical practice [3]. Its symptoms mainly include shoulder joint pain and limited joint movement, especially nocturnal pain. According to the location of supraspinatus tendon injury, Ellman divided it into articular-sided tears, bursal-sided tears, and intratendinous tears. According to the severity of tendon injury, it can be defined as grade I (≤ 3 mm), grade II (3–6 mm), and grade III (≥ 6 mm) [4, 5]. Ellman III partial articular-sided supraspinatus tendon tears are prone to further deterioration, and therefore, surgical treatment is generally recommended for these patients. Previous studies used open or minimally invasive open repair techniques for treatment [6]. However, at this stage, shoulder arthroscopy is usually used for treatment. At present, there are two common surgical methods for patients with severe articular-sided supraspinatus tendon tears, including the arthroscopic transtendon repair method and the arthroscopic full-thickness repair method [7, 8]. The arthroscopic full-thickness repair method converts partial tears into full-thickness tears for repair. It has the potential advantages of better access to the tendon footprint for preparation of the bony bed and removal of degenerative tissue [9]. But the surgery removes structurally sound bursal-sided tendon, which could increase the re-tear rate [10]. The arthroscopic transtendon repair method preserves the rotator cuff tissue on the bursal side and directly repairs the injured supraspinatus tendon in situ. Theoretically, it has two benefits: it preserves the intact part of the tendon and enhances its biomechanical characteristics (less gapping and higher mean ultimate failure strength) [9]. And it has been reported that some patients have slow recovery of the shoulder joint or residual discomfort in the shoulder joint after surgery, which may be related to the excessive tightening of the bursal side caused by the arthroscopic transtendon repair method [11–13]. However, currently, there are few studies comparing the clinical efficacy and related risk factors for the arthroscopic transtendon repair method and the arthroscopic full-thickness repair method in the treatment of severe articular supraspinatus tendon injury.

The purpose of this study was to retrospectively analyze the surgical treatment of PTRCT patients with Ellman III injuries in our hospital from 2017 to 2020 and to compare the postoperative therapeutic effects of the two arthroscopic surgery methods. The factors affecting the postoperative efficacy of the patients were investigated in this study. The purpose of this study is to provide guidance for clinical work.

## Methods

### General information

The research protocol of this retrospective study was approved by the local ethics committee. This study included PTRCT patients with Ellman III injuries who underwent surgical treatment in our hospital from January 2017 to January 2020. The inclusion criteria were as follows: (1) MRI results suggesting Ellman III injury, and only articular side tears of the supraspinatus tendon were confirmed by arthroscopy. The intratendinous and bursal sides of the supraspinatus tendon were intact; (2) conservative treatment failed for at least 3 months; (3) the surgical treatment method was the arthroscopic transtendon repair method or the arthroscopic full-thickness repair method; and (4) at least 24 months of follow-up. The exclusion criteria were as follows: (1) bursal-side tears of the supraspinatus tendon; (2) combined infraspinatus, teres minor or subscapular muscle injury; (3) previous history of ipsilateral shoulder surgery; (4) intraoperative glenoid labrum repair; and (5) missing data during follow-up.

### Patient information

A total of 84 PTRCT patients with Ellman III injuries were enrolled in this retrospective study. The surgical approach is based on the surgeon’s choice and preference. Among them, 32 patients underwent the arthroscopic transtendon repair method (Group A), and 52 patients underwent the arthroscopic full-thickness repair method (Group B). All study participants signed the informed consent form before surgery and agreed to undergo arthroscopic surgery, but they were unaware of the intraoperative procedure. In addition, informed consent was obtained from the participants for the data involved in this study.

### Surgical technique

The surgery was performed by the same senior doctor. All patients underwent brachial plexus block combined with systemic tracheal intubation, and blood pressure was controlled to 90 ∼ 110/50 ∼ 70 mmHg. The contralateral side was placed in a decubitus position, and the body was fixed in the posterior leaning position at 25°. Routine disinfection and draping were performed. Posterior access was first established to examine the glenohumeral joint. The synovial membrane in the glenohumeral joint was cleaned using a shaver and radiofrequency, and the long head of the biceps brachii tendon, the glenoid labrum, the glenohumeral ligament, the subscapular muscle, and the supraspinatus tendon were explored and evaluated. Then, the tear site of the supraspinatus tendon was found and observed. Arthroscopy was performed through the same posterior incision to enter the subacromial space in the direction of the anterolateral acromion, and the subacromial capsule was removed to expose the bursal side of the supraspinatus tendon and the footprint area. The integrity and texture of the rotator cuff tissue were evaluated with a probe. The surgeon used either of the two techniques for repair based on the patient’s age, activity level, and severity of the rotator cuff injury. Biceps tenotomy should be performed when the long head of the biceps tendon is unstable or the tendon is severely injured. Acromioplasty should be performed only when osteophytes are found below the acromion or the acromion is hook-shaped. The number of suture anchors was determined based on the anteroposterior length of the tearing tendon.

**Fig. 1:**
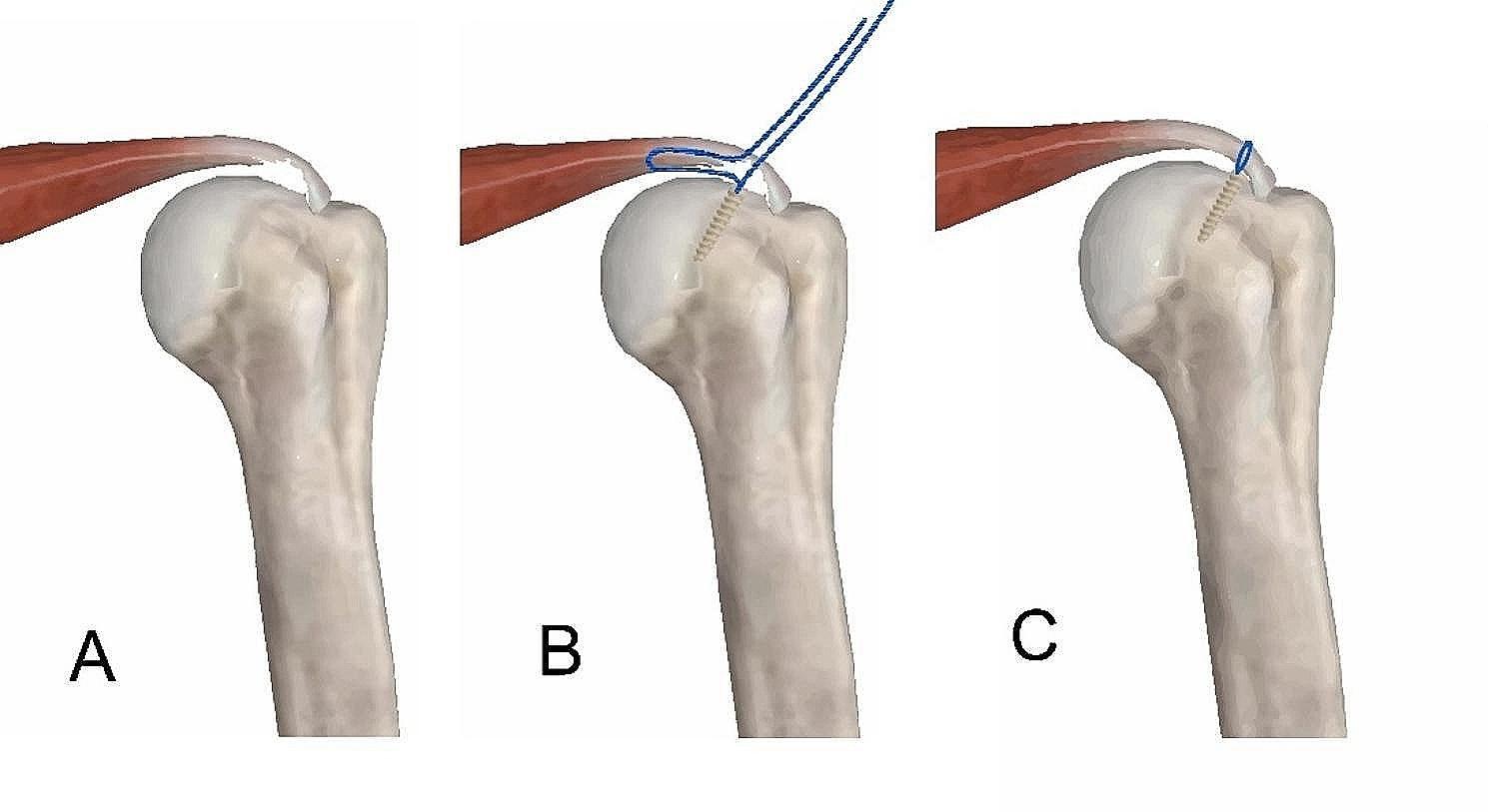
Arthroscopic fixation of the partially injured supraspinatus tendon. (**A**) The manifestations of partial injury of the supraspinatus tendon; (**B**) Complete placement of the absorbable anchor; (**C**) Complete arthroscopic trans-tendon fixation

Arthroscopic transtendon repair method (Fig. 1): The arthroscope was placed into the glenohumeral joint again, and a 4.5 mm full radius shaver was used to clean the degenerative tissue on the articular side of the supraspinatus tendon. Then, the surrounding bone was freshened and drilled. A gray trocar was used for percutaneous puncture at the anterolateral corner of the acromion to locate the anchor insertion point and angle. An 18-gauge spinal needle was inserted percutaneously through the tear, and a PDS suture (No.0 polydioxane) was introduced into the joint cavity. A suture grasper was used in the anterior superior approach to the space between the biceps longus tendon and the supraspinatus tendon joint capsule, and the corresponding sutures and PDS sutures were grasped. The arthroscope was placed in the subacromial space again, sutures of the same color were successively removed with the thread grasper, and knots were performed under the arthroscope. Finally, the arthroscope was placed into the glenohumeral joint to observe the reconstruction of the articular rotator cuff footprint.

**Fig. 2:**
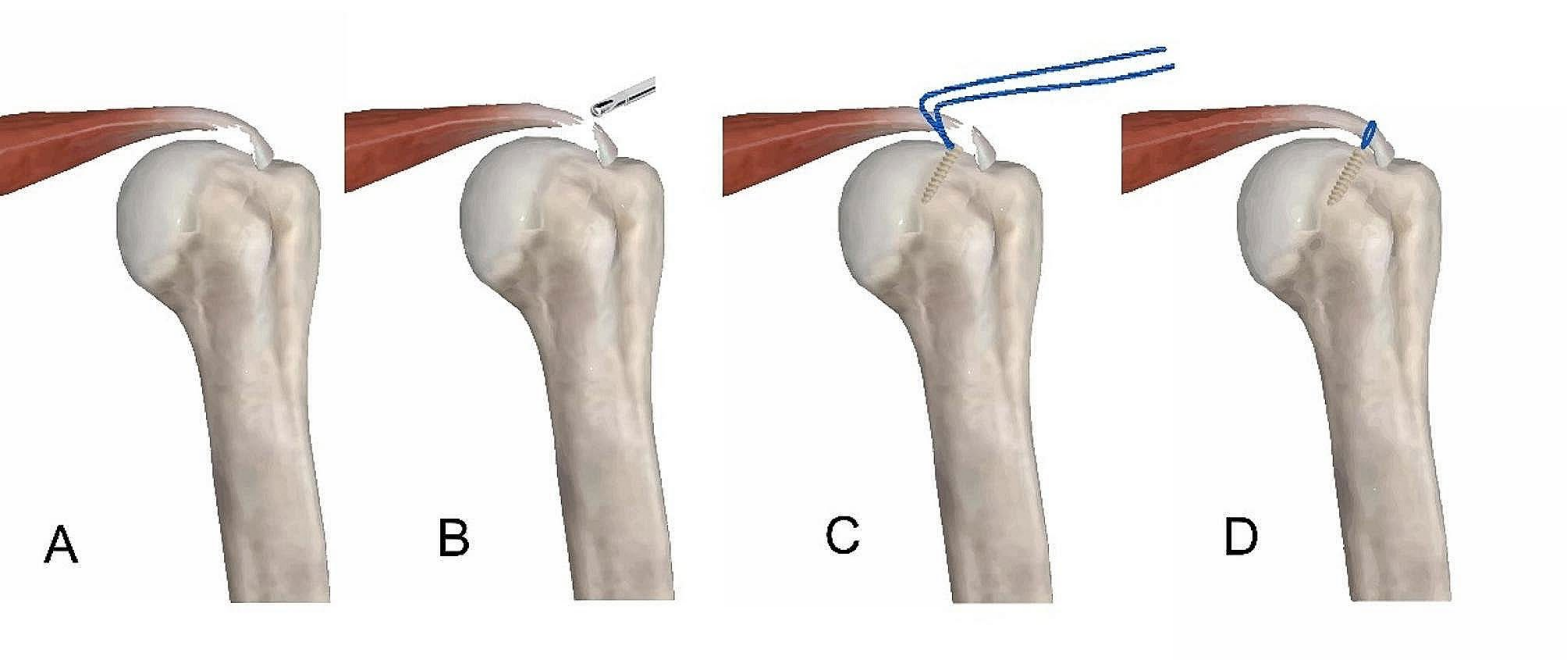
Partial injury of the supraspinatus tendon was converted to complete lesions for repair. (**A**) The manifestation of partial injury of the supraspinatus tendon; (**B**) The use of a shaver to convert the partial injury into a full-thickness injury; (**C**) Complete placement of the absorbable anchor; (**D**) Complete full-thickness repair of the injured tendon

Arthroscopic full-thickness repair method (Fig. 2): The arthroscopic approach enters the subacromial space through the posterior approach to locate the position of the PDS suture. Radiofrequency was used to open and clean the footprint area of the bursal side of the supraspinatus tendon, followed by a grinding drill to freshen the surrounding bone. A gray trocar was used for percutaneous puncture at the anterolateral corner of the acromion to locate the anchor insertion point and angle. The arthroscope was again placed into the glenohumeral joint to observe the reconstruction of the rotator cuff on the articular side.

### Postoperative rehabilitation

The patient continued to wear a shoulder abduction brace after surgery. Within 4 weeks after the surgery, the patients underwent limited passive function exercise with the brace. After 4 weeks, limited passive function exercise of the shoulder joint was performed after the brace was removed. These activities gradually increased at 8 weeks after the surgery, and muscle resistance exercise began at 12 weeks after the surgery.

### Evaluation indicators

All patients were followed up for at least 2 years. The Constant-Murley shoulder score (Constant score), American Society of Shoulder and Elbow Surgeons score (ASES score) and visual analog scale (VAS score) were used to evaluate the shoulder pain and function of patients before surgery, 6 months after surgery, and at the last follow-up. The ROM of the shoulder joint was measured with a goniometer before surgery, 6 months after surgery, and at the final follow-up, including forward flexion (FF), external rotation (ER), and internal rotation (IR). Postoperative satisfaction was obtained from patient self-assessment at the last follow-up. Data was recorded by two rehabilitation doctors who did not understand the surgical process, and the average of the two measurements was considered the measurement value.

### Statistical methods

All data calculations and statistical analyses were performed using SPSS 19.0 statistical software (IBM, Armonk, NY, USA). Numerical variables are expressed as the mean ± standard deviation, categorical data were compared using Pearson’s chi-square test, and continuous variables were compared using Student’s t test. Screening was performed according to whether the postoperative efficacy was satisfactory, and the risk factors were screened by univariate analysis. Then, binary logistic regression analysis was performed on the selected risk factors. *P* < 0.05 was considered significant in all cases.

## Results

**Fig. 3:**
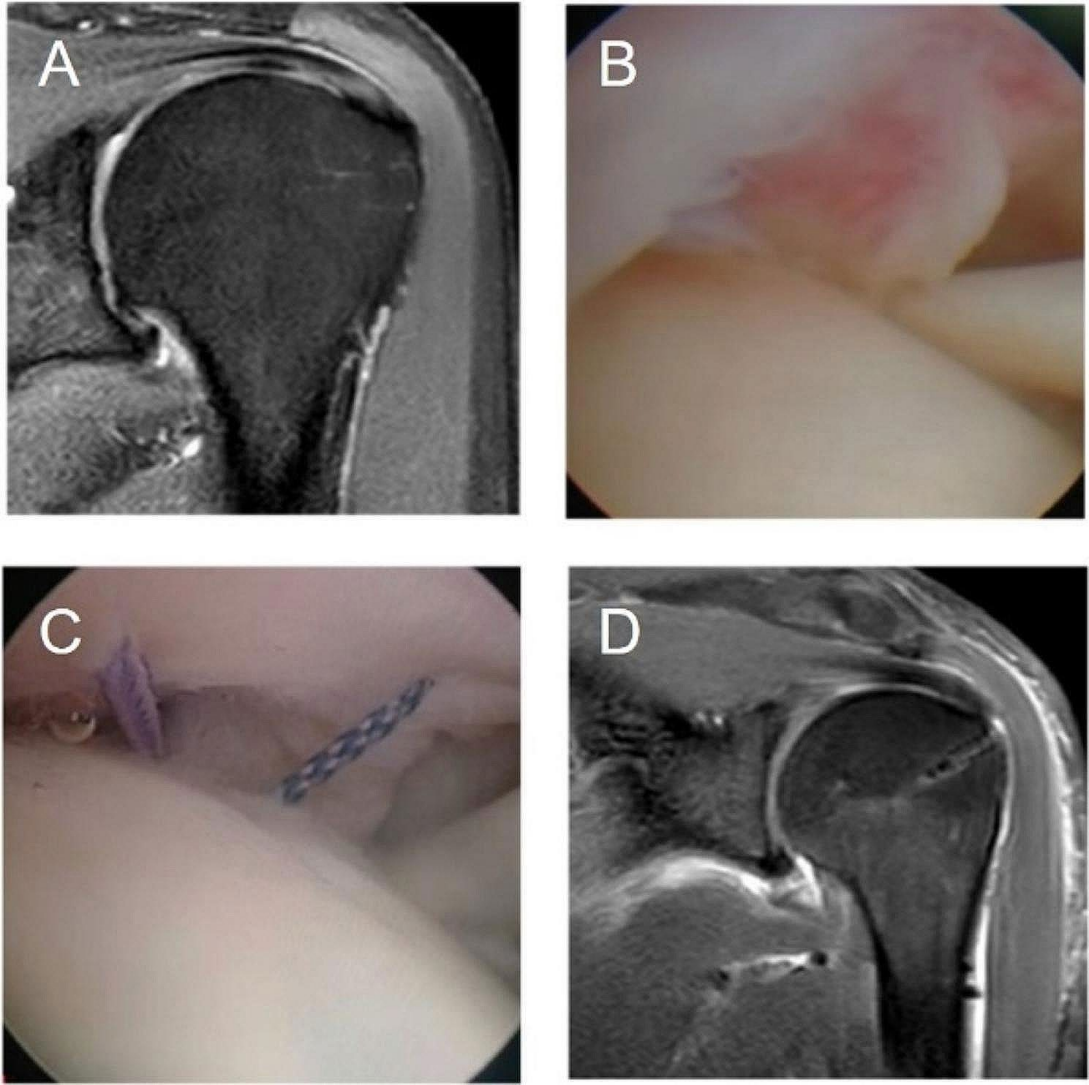
The patient underwent the arthroscopic trans-tendon repair method (**A**) Preoperative magnetic resonance imaging showed an Ellman III partial articular-sided supraspinatus tendon tear; (**B**) Partial tear of the supraspinatus tendon at the articular side in the glenohumeral joint; (**C**) Sutures were introduced into the glenohumeral joint; (**D**) Postoperative magnetic resonance imaging indicated satisfactory repair of the rotator cuff

**Fig. 4:**
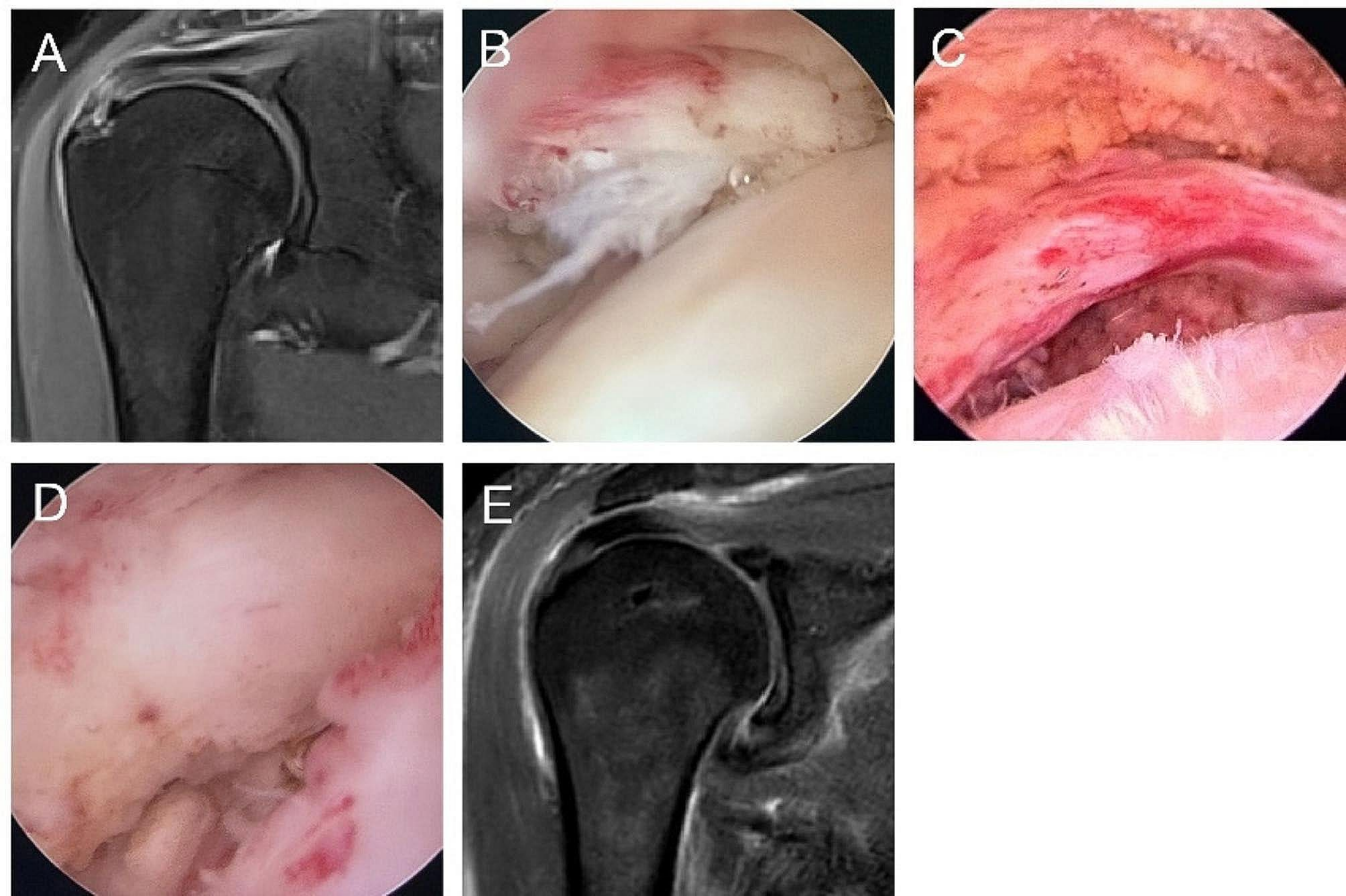
The patient underwent the arthroscopic full-thickness repair method (**A**) Preoperative magnetic resonance imaging showed Ellman III partial articular-sided supraspinatus tendon tear; (**B**) Partial tear of the supraspinatus tendon at the articular side in the glenohumeral joint; (**C**) Partial injury of supraspinatus tendon transforms into a full-thickness injury; (**D**) The supraspinatus tendon was effectively repaired within the glenohumeral joint; (**E**) Postoperative magnetic resonance imaging indicated satisfactory repair of the rotator cuff

A total of 84 PTRCT patients met the inclusion criteria. Among them, 32 patients underwent the arthroscopic transtendon repair method, and 52 patients underwent the arthroscopic full-thickness repair method. Preoperative and postoperative magnetic resonance images and intraoperative conditions of a patient undergoing the arthroscopic transtendon repair method and a patient undergoing the arthroscopic full-thickness repair method are shown in Figs. 3 and 4. Thirty-two patients (38.1%) underwent biceps tenotomy due to tendon instability or severe damage to the long head of the biceps; 21 patients (25%) underwent acromioplasty due to type II or type III acromion. The general information of the enrolled patients is shown in Table 1.

**Table 1:** Demographic data of the included patients

	Trans-tendon group	Completion tear group	*P* value
Age(yr)^*^	55.4 ± 6.7	58.8 ± 7.3	0.522
Smoke	6	16	0.168
Sex(M/F)	10/22	17/35	0.785
Diabetes	7	12	0.8
Symptom duration(mo)^*^	6.2 ± 2.5	7.4 ± 2.4	0.599
Acromioplasty	9	12	0.319
Biceps tenotomy	13	19	0.484

*Data are reported as mean ± standard error of mean

### Within-group analysis

The Constant scores, ASES scores, and VAS scores of the two groups of patients before surgery, 6 months after surgery, and at the final follow-up were all improved compared with those before surgery, and the differences were statistically significant (*P* < 0.05). The FF, ER, and IR of the patients in the arthroscopic transtendon repair group at 6 months after surgery and at the last follow-up were significantly higher than those before surgery (*P* < 0.05). The FF of the patients in the arthroscopic full-thickness repair group at 6 months after surgery and at the final follow-up was significantly higher than that before surgery (*P* < 0.05), while the ER and IR at 6 months after surgery were not significantly higher than those before surgery. See Table 2.

**Table 2:** Comparison of shoulder joint pain and function of patients in the arthroscopic transtendon repair group and the arthroscopic full-thickness repair group before surgery, 6 months after surgery, and at the last follow-up

	Preoperatively	6 mo postoperatively	Final follow-up
Trans-tendon group			
Constant score	49.9 ± 6.2	63.3 ± 5.7^#^	77.5 ± 5.7^%^
ASES score	50.8 ± 6.6	63.6 ± 5.3^#^	78.4 ± 6.7^%^
VAS score	5.5 ± 1.2	4.2 ± 0.9^#^	2.2 ± 1.1^%^
FF(°)	105.4 ± 10.5	124.6 ± 16.3^#^	144.8 ± 19.4^%^
ER(°)	45.3 ± 5.7	52.1 ± 5.8^#^	58.6 ± 10.0^%^
IR(°)	27.2 ± 8.4	34.1 ± 10.2^#^	43.5 ± 11.7^%^
Completion tear group			
Constant score	47.5 ± 5.7	62.2 ± 5.8^#^	75.2 ± 7.4^%^
ASES score	48.9 ± 6.1	63.6 ± 5.3^#^	76.1 ± 7.2^%^
VAS score	5.7 ± 1.1	3.9 ± 0.9^#^	2.2 ± 1.0^%^
FF(°)	100.9 ± 9.1	124.2 ± 10.6^#^	140.1 ± 17.5^%^
ER(°)	46.1 ± 5.1	48.9 ± 7.3	59.6 ± 9.2^%^
IR(°)	29.8 ± 7.2	34.8 ± 7.4	41.9 ± 9.5^%^

NOTE. Data are reported as mean ± standard error of mean

Abbreviations: FF: forward flexion; ER: external rotation at side; IR: internal rotation

#Statistically significant difference between the preoperatively group and 6 mo postoperatively group (*P*<0.05)

%Statistically significant difference between the preoperatively group and final follow-up group (*P*<0.05)

### Intergroup analysis

There was no significant difference in the Constant score, ASES score, or VAS score between the patients in the arthroscopic transtendon repair group and the arthroscopic full-thickness repair group before surgery, 6 months after surgery, or at the last follow-up (*P* > 0.05) (*P* > 0.05). There was no statistically significant difference in ER or IR between the two groups before surgery, 6 months after surgery, or at the last follow-up (*P* > 0.05). There was a significant difference in FF between the two Groups 6 months after surgery (*P* = 0.003). See Table 3.

**Table 3:** Comparison of shoulder joint pain and function between patients in the arthroscopic transtendon repair group and the arthroscopic full-thickness repair group before surgery, 6 months after surgery, and at the last follow-up

	Trans-tendon group	Completion tear group	*P* value
Constant score			
Preoperatively	49.9 ± 6.2	47.5 ± 5.7	0.408
6 mo postoperatively	63.3 ± 5.7	62.2 ± 5.8	0.527
Final follow-up	77.5 ± 5.7	75.2 ± 7.4	0.332
ASES score			
Preoperatively	50.8 ± 6.6	48.9 ± 6.1	0.668
6 mo postoperatively	63.6 ± 5.3	63.6 ± 5.3	0.375
Final follow-up	78.4 ± 6.7	76.1 ± 7.2	0.84
VAS score			
Preoperatively	5.5 ± 1.2	5.7 ± 1.1	0.663
6 mo postoperatively	4.2 ± 0.9	3.9 ± 0.9	0.90
Final follow-up	2.2 ± 1.1	2.2 ± 1.0	0.768
FF(°)			
Preoperatively	105.4 ± 10.5	100.9 ± 9.1	0.178
6 mo postoperatively	124.6 ± 16.3	124.2 ± 10.6	0.003
Final follow-up	144.8 ± 19.4	140.1 ± 17.5	0.634
ER(°)			
Preoperatively	45.3 ± 5.7	46.1 ± 5.1	0.722
6 mo postoperatively	52.1 ± 5.8	48.9 ± 7.3	0.120
Final follow-up	58.6 ± 10.0	59.6 ± 9.2	0.538
IR(°)			
Preoperatively	27.2 ± 8.4	29.8 ± 7.2	0.284
6 mo postoperatively	34.1 ± 10.2	34.8 ± 7.4	0.078
Final follow-up	43.5 ± 11.7	41.9 ± 9.5	0.196

NOTE. Data are reported as mean ± standard error of mean

Abbreviations: FF: forward flexion; ER: external rotation at side; IR: internal rotation

### Multivariate regression analysis

A univariate analysis of postoperative satisfaction was performed on the 84 enrolled patients. There were no statistically significant differences in age, sex, smoking status, history of diabetes, duration of symptoms, or surgical methods between the satisfactory group and the dissatisfied group (*P* > 0.1). Univariate analysis showed that whether acromioplasty or biceps tenotomy was performed, preoperative ASES score and preoperative VAS score may be risk factors related to postoperative satisfaction (*P* < 0.1). See Table 4. The risk factors selected by univariate analysis were subjected to binary logistic regression analysis. The results showed that the preoperative ASES score and whether biceps tenotomy was performed were independent risk factors for satisfactory postoperative efficacy (*P* < 0.05). See Table 5.

**Table 4:** Results of univariate analysis of satisfactory postoperative outcomes in PTRCT patients with Ellman III injuries

Variate	Dissatisfied group	Satisfactory group	*P* value
Age(y)	58.1 ± 7.9	57.1 ± 6.7	0.509
Symptom duration(mo)	7.4 ± 2.5	6.6 ± 2.4	0.166
Preoperative ASES score	53.4 ± 7.1	47.0 ± 4.1	0.001
Preoperative VAS score	4.9 ± 1.2	6.1 ± 0.8	0.001
Sex[case(%)]			
Male	9(26.5)	18(36)	
Female	25(73.5)	32(64)	0.365
Smoke[case(%)]			
Yes	7(20.6)	16(32)	
No	27(79.4)	34(68)	0.255
Diabetes[case(%)]			
Yes	7(20.6)	12(24)	
No	27(79.4)	38(76)	0.718
Acromioplasty[case(%)]			
Yes	5(14.7)	16(32)	
No	29(85.3)	34(68)	0.074
Biceps tenotomy[case(%)]			
Yes	9(26.5)	23(46)	
No	25(73.5)	27(54)	0.072
Surgical method[case(%)]			
Trans-tendon	11(32.4)	21(42)	
Completion tear	23(67.6)	29(58)	0.378

NOTE. Data are reported as mean ± standard error of mean

Abbreviations: Univariate analysis screened out risk factors related to postoperative satisfaction(*P*<0.1)

**Table 5:** Results of binary logistic regression analysis of satisfactory postoperative efficacy in PTRCT patients with Ellman III injuries

	Coefficient	OR	95%CI	*P* value
Preoperative ASES score	-2.93	0.053	0.015 ∼ 0.185	0.001
Biceps tenotomy	1.70	5.423	1.49 ∼ 19.7	0.01

NOTE. OR, odds ration; CI, confidence interval

## Discussion

The arthroscopic technique of shoulder surgery is growing increasingly popular in the clinical treatment of rotator cuff disorders. For patients with severe partial articular-sided supraspinatus tendon tears, the most common surgical methods include the arthroscopic transtendon repair method and the arthroscopic full-thickness repair method [13]. Pursuing tendon-bone healing of the rotator cuff tendon after surgery is a major goal of clinical treatment. Tendon-bone healing can indeed achieve a better clinical outcome, but the failure of tendon-bone healing or retear does not completely mean the failure of clinical treatment [15]. Patients are more concerned with decreased postoperative pain and functional improvement. Postoperative pain and functional improvement can significantly improve the postoperative satisfaction of patients. Therefore, how to preevaluate the postoperative efficacy of patients before surgery and develop a more targeted treatment plan have become the focus of research in recent years.

This study compared the efficacy of two surgical methods in the treatment of PTRCT patients with Ellman III injuries. The follow-up results showed that both achieved good clinical outcomes. The results showed that postoperative pain and function were significantly improved. However, a comparison of patients who underwent arthroscopic transtendon repair and arthroscopic full-thickness repair showed that there was no significant difference in the improvement of postoperative pain and function. And there is no consensus in the literature on the best technique for repairing PTRCT patients with Ellman III injuries. Castagna [1] randomly divided 72 patients into the arthroscopic transtendon repair group and the arthroscopic full-thickness repair group. The results showed that the two techniques achieved satisfactory results in terms of function and pain, and there was no significant difference between the two groups. Shin [12] conducted a prospective comparison between the tendon integrity potential and clinical outcomes of these two surgical approaches. The results showed that both surgical techniques might result in satisfactory functional improvements and pain relief. These results are consistent with a portion of the findings of our study. Therefore, we suggest that surgeons can choose the appropriate surgical method according to their habits and skill level.

The arthroscopic transtendon repair method preserves the bursal-side supraspinatus tendon footprint and anatomically reconstructs the internal measurement footprint [16]. The intact bursal-side tendon protects the repaired articular-side tendon, with a low incidence of retear and long-term clinical outcomes [9]. However, this treatment method is very difficult. When the injured tendon on the side of the joint is severely retracted, it easily causes excessive tension of the repaired tendon, resulting in a mismatch of tendon length and tension, leading to increased early postoperative pain and postoperative functional limitations [9, 17]. According to some clinical researches, the arthroscopic transtendon repair method is associated with an increased risk of postoperative shoulder stiffness [17, 18]. Shin found that the range-of-motion of the shoulder joint was slow to recover after the arthroscopic transtendon repair method, and three patients developed adhesive capsulitis at 6 months after surgery [12]. Huberty reported 5% of patients with adhesive capsulitis after transtendon repair and requiring reoperation for postoperative stiffness [17]. This method has the danger of producing nonphysiologic tension in the remaining fibers and overtightening the bursal area of the cuff. The source of the shoulder stiffness could be this changed tensioning on both sides of the rotator cuff tendons [19]. Similar results were observed in our study, where early shoulder stiffness was more common in patients after the transtendon repair method. The arthroscopic full-thickness repair method is easy to master, and the operation time is relatively short, which can reduce postoperative inflammation [20]. However, this method requires the surgeon to cut off the normal supraspinatus tendon tissue of the bursa in the footprint area, which makes it difficult to anatomically reconstruct the footprint area [4, 12]. And turning a partial lesion into a complete lesion that misaligns the entire lateral edge of the rotator cuff leads to the risk of a nonanatomic repair that alters biomechanics, which can lead to early tendon degeneration [21]. This method eventually causes scar healing between the tendon and the bone tissue in the footprint area. Sun performed a meta-analysis comparing the two surgical techniques and showed that the arthroscopic full-thickness repair group had a higher rate of postoperative re-tears [8]. Overall, the above two surgical methods have their own advantages and disadvantages, and the efficacy (pain and function) of surgical patients has been significantly improved.

There are many factors that affect the postoperative satisfaction of patients, and factors such as patient age, smoking status, and diabetes mellitus have significant effects on postoperative rotator cuff tendon healing [20, 22]. Age is an inexorable factor that also affects the elastic modulus of the rotator cuff through the natural process of tendon degeneration. And younger patients usually have high functional demands after surgery and may participate more in activities of daily living and sports, which has a significant impact on postoperative satisfaction. Nicotine is known to affect the expression of MMP-9 in tendon cells, which leads to modification of the modulus of elasticity of the tendon, and Park’s study showed that smoking is an important risk factor for the spread of lesions [23]. In relation to the impact of diabetes mellitus on the prognosis of cuff repair, it is recognized that the likelihood of non-healing of rebuilt tendons increases if hyperglycemia persists throughout the postoperative phase [24]. In this study, PTRCT patients with Ellman III injuries who underwent shoulder arthroscopy for treatment were included. The effects of various factors, such as age, sex, and surgical approach, on postoperative satisfaction were compared. However, differences in age, smoking status, and history of diabetes mellitus were not found to have a significant impact on patient postoperative satisfaction in our study. The preoperative ASES score and whether biceps tenotomy was performed were independent risk factors for satisfactory postoperative efficacy. Patients with a low preoperative ASES score showed a high degree of preoperative pain and functional limitation. After receiving shoulder arthroscopy, postoperative pain and function usually effectively improved, and the postoperative satisfaction of the patients was high. During the active and passive movement of the shoulder joint, the long head of the biceps tendon repeatedly slides in the sulcus between the large and small nodules, and long-term repeated sliding friction will lead to aggravation of tendon inflammation [7, 25]. Head tendinitis is closely related to tendon inflammation. For older patients, postoperative biceps tendon tenotomy can often effectively relieve postoperative pain and improve postoperative satisfaction [26]. Preassessment of the pain and function of the shoulder joint of the patient before the surgery and the development of an accurate treatment plan can lead to a good degree of satisfaction for the patient after the surgery.

This study has several limitations. First, this study is a single-center retrospective study with a limited sample size and short follow-up time. There are other factors that may interfere with the comparative study results. Multicenter cooperation is needed to collect more cases and extend the follow-up time. Second, there were certain errors in the evaluation and measurement of postoperative pain, function, and mobility, which may have a certain impact on the study results.

## Conclusion

This study compared the efficacy of the arthroscopic transtendon repair method and the arthroscopic full-thickness repair method in the treatment of PTRCT patients with Ellman III injuries. However, there was no significant difference in efficacy between the two surgical methods. Surgeons should choose the appropriate surgical method according to their own habits and skill level. The preoperative ASES score and whether biceps tenotomy was performed were independent predictors of satisfactory postoperative efficacy in PTRCT patients with Ellman III injuries.

### Electronic supplementary material

Below is the link to the electronic supplementary material.

Supplementary Material 1

## Data Availability

The raw data is provided in the item of supplementary file.
